# New Insights of Salicylic Acid Into Stamen Abortion of Female Flowers in Tung Tree (*Vernicia fordii*)

**DOI:** 10.3389/fgene.2019.00316

**Published:** 2019-04-05

**Authors:** Meilan Liu, Wenying Li, Guang Zhao, Xiaoming Fan, Hongxu Long, Yanru Fan, Mingwang Shi, Xiaofeng Tan, Lin Zhang

**Affiliations:** ^1^Key Laboratory of Cultivation and Protection for Non-Wood Forest Trees, Ministry of Education, Central South University of Forestry and Technology, Changsha, China; ^2^Key Lab of Non-wood Forest Products of State Forestry Administration, College of Forestry, Central South University of Forestry and Technology, Changsha, China; ^3^Henan Institute of Science and Technology, Xinxiang, China

**Keywords:** tung tree, salicylic acid, programmed cell death, flower development, unisexual flowers, stamen abortion

## Abstract

Tung tree (*Vernicia fordii*), an economically important woody oil plant, is a monoecious and diclinous species with male and female flowers on the same inflorescence. The extremely low proportion of female flowers leads to low fruit yield in tung orchards. The female flower normally develops along with stamen abortion; otherwise sterile ovules will be produced. However, little knowledge is known about the molecular basis of the female flower development in tung tree. In this study, integrated analyses of morphological and cytological observations, endogenous phytohormone assay and RNA-seq were conducted to understand the molecular mechanism of the female flower development in tung tree. Cytological observation suggested that the abortion of stamens in female flowers (SFFs) belongs to the type of programmed cell death (PCD), which was caused by tapetum degeneration at microspore mother cell stage. A total of 1,366 differentially expressed genes (DEGs) were identified in female flowers by RNA-seq analysis, of which 279 (20.42%) DEGs were significantly enriched in phenylpropanoid biosynthesis, phenylalanine metabolism, flavonoid biosynthesis, starch and sucrose metabolism, and plant hormone signal transduction. Stage-specific transcript identification detected dynamically expressed genes of important transcription regulators in female flowers that may be involved in PCD and floral organ development. Gene expression patterns revealed that 17 anther and pollen development genes and 37 PCD-related genes might be involved in the abortion of SFF. Further analyses of phytohormone levels and co-expression networks suggested that salicylic acid (SA) accumulation could trigger PCD and inhibit the development of SFF in tung tree. This study provides new insights into the role of SA in regulating the abortion of SFF to develop normal female flowers.

## Introduction

Flower development attracts great attention as a fascinating topic for studying plant development and evolution. Unisexuality is considered to be an important transition in the evolutionary history of angiosperms (Barrett, 2010). Many flowers become unisexual after floral organs are specified, but during the process of differentiation, carpel or stamen abortion or arrest occurs and organs become non-functional (Sobral et al., 2016). Plant reproduction requires the development of complex structures that interact with each other and that have an inherently limited life span. Thus, programmed cell death (PCD) is involved in shaping the sexual and non-sexual organs of the flower and in their removal once they are no longer needed (Wu and Cheun, 2000). For example, the gynoecium in male flowers is degenerated by PCD just after initiation of floral primordium in *Zea mays* (Cheng et al., 1983). In *Actinidia deliciosa*, PCD also induces male sterility in female flowers (Coimbra et al., 2004).

Researchers suggest that PCD has a close relationship with the accumulation of salicylic acid (SA), and SA controls the timing and extent of PCD in the hypersensitive response (HR) (Greenberg, 1997; Alvarez, 2000; Kovacs et al., 2016). As an endogenous signaling molecule, however, SA is suggested in connection with plant flowering. For example, SA induces the expression of FLOWERING LOCUST 2 (FT2) in the regulation of the flowering in *Pharbitis nil* (Yamada and Takeno, 2014). In *Arabidopsis*, SA controls early flower development by NON-EXPRESSOR OF PR3 (NPR3) (Shi et al., 2013). Moreover, SA plays an important role in regulating pollen viability and floret fertility in rice (Zhao et al., 2018). SA can also induce mRNA accumulation of Callose synthase 5 (Gsl5) for deposition of callose in pollens (Ostergaard et al., 2002).

Tung tree (*Vernicia fordii*), native to China, is widely planted in China and other countries for its ornamental purpose and tung oil production (Cui et al., 2018; Li et al., 2018). Tung oil shows strong dryness, strong adhesion, acid and alkali resistance, so it has been widely used in various industrial fields (Cahoon et al., 1999; Chen et al., 2010). In recent years, tung oil has been attracted world-wide attention due to production security, environmental concerns, and negative effect of synthetic chemical coatings on human health (Meininghaus et al., 2000; Tsakas et al., 2011; Wei et al., 2012; Yang et al., 2018). As a monoecious plant species, tung tree produces a low ratio of female to male flowers (approximately 1:27) (McCann, 1942) and functionally abnormal female flowers, which results in low fruit yield in tung orchards. Generally, female tung flowers present bisexual characteristics at early stages, and then switch to unisexual flowers at late stages along with cell death in stamens in female flowers (SFFs) (Mao et al., 2017). However, the reason causing cell death and the transition of tung flowers from bisexuality to unisexuality remains uncertain.

Mao et al. (2017) found that PCD occurred during stamen abortion in female tung flowers, whereas the molecular mechanism is unclear. Toward this end, we conducted integrated analyses of morphological and cytological observations, endogenous phytohormone assay and RNA-seq of male and female flowers. Based on these analyses, we proposed a possible mechanism for female flower development in tung tree.

## Materials and Methods

### Plant Materials

**FIGURE 1:**
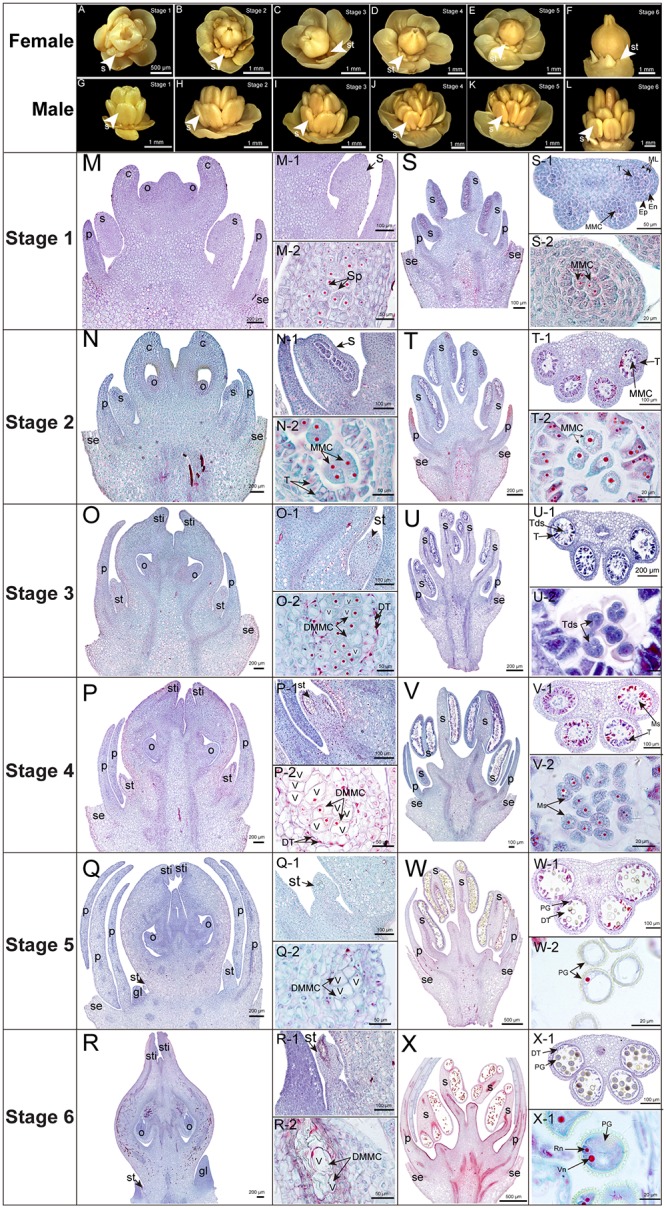
Morphological and cytological observations of male and female flowers. **(A–L)** Morphological observation. **(M–X)** Cytological observation. Stages 1–6, 35 DBF (stage 1), 30 DBF (stage 2), 25 DBF (stage 3), 20 DBF (stage 4), 15 DBF (stage 5), 10 DBF (stage 6); se, sepal; p, petal; s, stamen; st, staminode; c, carpel; o, ovule; sti, stigma; gl, gland; Sp, sporogenous cells; MMC, microspore mother cells; Tds, tetrads; Ms, microspore; Pg, pollen grains; Ep, epidermis; En, endothecium; ML, middle layer; T, tapetum; DMMC, degraded microspore mother cells; DT, degraded tapetum; V, vacuole; Vn, vegetative nucleus; Rn, reproduction nucleus.

The flower was collected from 6-year-old tung tree ‘Putaotong’ grown in the experimental area of the Central South University of Forestry and Technology (Qingping Town, Yongshun County, Hunan Province) under natural conditions. The flower was randomly collected every 5 days from March to April, 2017. Male and female flowers at stage 2 in 30 days before flowering (DBF, C1 and X1, Figure 1C,D), stage 4 in 20 DBF (C2 and X2, Figure 1G,H), stage 6 in 10 DBF (C3 and X3, Figure 1K,L) were separately collected from the same plant and used for transcriptome sequencing. Three biological replicates were analyzed for each sample with 100 flowers counted as one replicate. Controlled pollination (‘Putaotong’ × ‘Duiniantong’) was performed in April, 2017. A total of 300 female flowers and 300 bisexual flowers were used to evaluate the fruit setting with three replicates (each 100 flowers).

### Morphological and Cytological Observation

Morphology of male and female flowers at six developmental stages was observed by a 3D super depth digital microscope (ZEISS Smartzoom 5, Germany). The samples were prefixed and observed with the paraffin sectioning method described by Liao et al. (2014). The samples were sectioned into 8–10 μm sections using a Leica RM 2265 microtome (Leica Camera AG, Germany), and were stained with Safranin O and Fast Green FCF according to the Sass’s method (Ruzin, 2000). Observations and photomicroscope of the sections were conducted using a microscope (Olympus BX-51, Japan).

### RNA Extraction and Sequencing

Total RNAs were isolated using RNAprep pure plant kit DP441 (TIANGEN, China), according to the manufacturer’s protocol. RNA sequencing was performed by Illumina Hiseq 2000 (Illumina, United States). Our reference genome sequences and annotations of tung tree were appliced for the analysis of RNA-seq data. Raw reads of the RNA-seq data are found in NCBI Sequence Read Archive database with the Accession Nos. SRX3843588; SRS3089151; SRS3089154; SRS3089148; SRS3089147; and SRS3089150.

### Data Analysis

The detailed processes of *de novo* assembly and functional annotation were performed as described by Feng et al. (2017) The number of all mapped reads for each gene was counted and normalized into the Fragments Per Kilobase of transcript per Million fragments mapped (FPKM) (Florea et al., 2013). Next, DESeq (Anders and Huber, 2010) was used to identify differentially expressed genes (DEGs), and genes with an adjusted false discovery rate (FDR) ≤ 0.01 and log2^FoldChange^ absolute value of ≥ 1 were marked as significantly different between the two samples. Stage-specific genes were selected as described by Feng et al. (2017). GO and KEGG pathway analyses were performed, and for each category, a two-tailed Fisher’s exact test was employed to test the enrichment of the identified protein against all database proteins. Correction for multiple-hypothesis testing was performed using standard FDR-control methods. The venn diagram, clustering, heatmap, and principal component analysis (PCA) were performed with the venn diagram function, the k-means function, pheatmap, and factoextra package in R software, respectively.

### Co-expression Networks Analysis

The transcripts with an average abundance (calculated from three biological replicates) of ≥ 1 (FPKM value) in at least one of the six samples were utilized to construct a co-expression network using the weighted correlation network analysis (WGCNA) R software package (Langfelder and Horvath, 2008). Modules were constructed using the following parameters: maxBlockSize = 10000, power = 18, networkType = “unsigned,” mergeCutHeight = 0.25, minModuleSize = 30. The significant modules in female flowers were determined according to an effective threshold (≥0.85) of the Pearson’s correlation coefficient (PCC) value and a *p*-value of ≤ 0.05. In order to identify genes which were correlated in expression with those genes involved in anther and pollen development, PCD, and SA, the top 50 genes in each significant module were used for network construction according to correlation degree. The co-expression networks were visualized using the Cytoscape v.3.5.1 program^1^ (Shannon et al., 2003).

### Quantitative Real-Time PCR (qPCR) and Phytohormone Levels Analysis

A PrimeScript RT enzyme with a gDNA eraser (Takara, Japan) was used for cDNA synthesis. QPCR was performed on a Bio-Rad CFX96 Real Time PCR system using SYBR Premix ExTaq II (Takara, Japan). The primers in this step were listed in Supplementary Table S1. Tung *Elongation Factor 1-α* (*EF1α*) was used as the internal control (Han et al., 2012). The relative expression levels were calculated using the 2^-ΔΔCT^ method (Livak and Schmittgen, 2001). Phytohormone levels were analyzed using the methods described by Pan et al. (2002).

### Statistical Analysis

All experiments were performed with three biological repeats. The data were analyzed by one-way ANOVA procedure in SPSS 22.0 (IBM Corporation, United States). All figures showed the average value of three repeats. All data were expressed as means plus or minus standard deviations (mean ± SD).

## Results

### Tapetum Degeneration at Microspore Mother Cell Stage Induces PCD and Abortion of SFF

Tung tree is a diclinous species and the functionally normal female flower develops along with stamen abortion in tung tree (Mao et al., 2017). However, the development of bisexual flowers often occurs in inflorescence with abnormal ovaries, leading to lower fruiting ratio (∼5%) than normal female flowers (∼68%) (Supplementary Figure S1). Therefore, we focused on staminode development in female flowers aiming to clarify the process of stamen abortion in tung tree. The development of female flowers could be divided into six important stages (stages 1–6) (Figure 1A–F). Female flowers possessed obvious stamens at stages 1 and 2 which initially degenerated from stage 3 and completely disappeared until stage 6 (Figure 1A–F), whereas no pistil was observed and only intact stamen developed in male flowers across all the development stages (Figure 1G–L).

The SFF showed delayed development in comparison with stamens in male flowers (SMFs). The SFF developed into sporogenous cell stage at 35 DBF (stage 1) (Figure 1M), while the SMF developed into early microspore mother cell (MMC) stage (Figure 1S). When the SFF developed into early MMC stage at 30 DBF (stage 2) (Figure 1N), the SMF developed into later MMC stage (Figure 1T). Interestingly, the SFF stopped growth at early MMC stage with tapetum cells degeneration and large vacuoles in MMC at 25 DBF (stage 3) (Figure 1O), while the SMF developed into tetrads stage (Figure 1U). From 20 to 10 DBF (stages 4 to 6), the SFF shrank at carpel base, and the MMC was mostly occupied by vacuoles with nucleuses in MMC disappearing (Figure 1P–R), while the SMF developed into binuclear pollen stage (Figure 1V–X).

Taken together, the functionally normal female flower developed along with stamen abortion in tung tree. The SFF developed normally at stages 1 and 2, but degenerated from stages 3 to 6. Our morphological and cytological observation further confirmed that the abortion of SFF in tung tree belongs to the type of PCD. The tapetum cell degeneration should contribute to the abortion of SFF, finally leading to PCD in MMC.

### DEGs in Male and Female Flowers

In order to elucidate the molecular mechanism responsible for the abortion of SFF in tung tree, the male and female flowers at stage 2 (X1, C1), stage 4 ( X2, C2), and stage 6 (X3, C3) were RNA-sequenced. Based on our tung tree genome sequence, a total of 22,344 genes were identified, of which 18,334 genes were expressed (FPKM value ≥ 1) across six flower samples.

**FIGURE 2:**
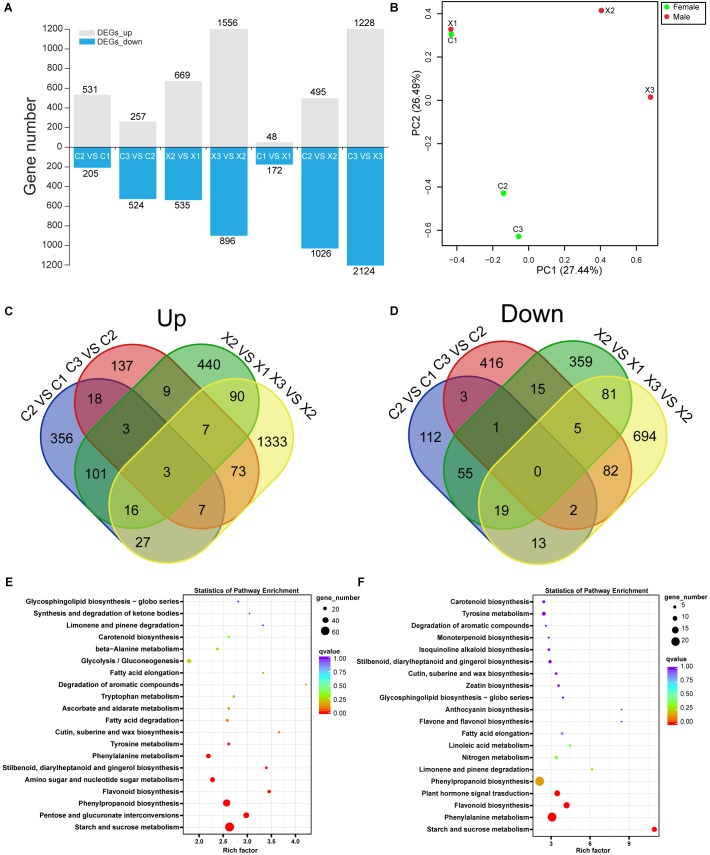
Analysis of DEGs in male and female flowers. **(A)** Numbers of DEGs in male and female flowers. **(B)** PCA plot of DEGs in male and female flowers. **(C,D)** Venn diagrams of the up-regulated and down-regulated DEGs in male and female flowers. **(E,F)** KEGG enrichment of DEGs in male and female flowers.

Further, we identified 3,147 DEGs in male flowers (X2_vs_X1, X3_vs_X2) and 1,366 DEGs in female flowers (C2_vs_C1, C3_vs_C2) (Figure 2A). The PCA analysis of the total DEGs, revealed that X1 and C1 had high correlation (Figure 2B), which indicating that the SFF in C1 had normal structure and function as MFF in X1. Besides, among the 1,366 DEGs in female flowers, 531 and 257 genes were significantly up-regulated in C2 and C3, respectively, and 205 and 524 genes were significantly down-regulated in C2 and C3, respectively, as compared to their preceding stage (Figure 2A). Furthermore, in C2_vs_C1 group, there were 356 specific DEGs among up-regulated genes, but 112 among down-regulated genes. In C3_vs_C2 group, 137 specific DEGs were up-regulated and 416 specific DEGs were down-regulated. These results showed that there were more genes highly expressed in C2 than C1 and C3, indicating that a large number of genes might participate in the transition from bisexual to unisexual flowers formation (Figure 2C,D).

KEGG annotation showed that 496 (15.76%) DEGs in male flowers were enriched in 110 KEGG pathways (Supplementary Table S2), of which the starch and sucrose metabolism, phenylpropanoid biosynthesis, pentose and glucuronate interconversions, flavonoid biosynthesis, amino sugar and nucleotide sugar metabolism were significantly enriched pathways (Figure 2E). In contrast, a total of 279 (20.42%) DEGs in female flowers were enriched in 103 KEGG pathways (Supplementary Table S2), of which the phenylpropanoid biosynthesis, phenylalanine metabolism, flavonoid biosynthesis, starch and sucrose metabolism, plant hormone signal transduction were significantly enriched pathways (Figure 2F). It has been reported that sucrose accumulation can change hormonal levels in cut lily flowers and phenylpropanoid can provide the key precursor for SA biosynthesis (Mauchmani and Slusarenko, 1996; Arrom and Munne-Bosch, 2012). In this study, 13 DEGs were found to be significantly enriched in plant hormone signal pathway, indicating that the phytohormones may play an important role in regulating the development of female flowers in tung tree.

### Stage-Specific Genes in Male and Female Flowers

**FIGURE 3:**
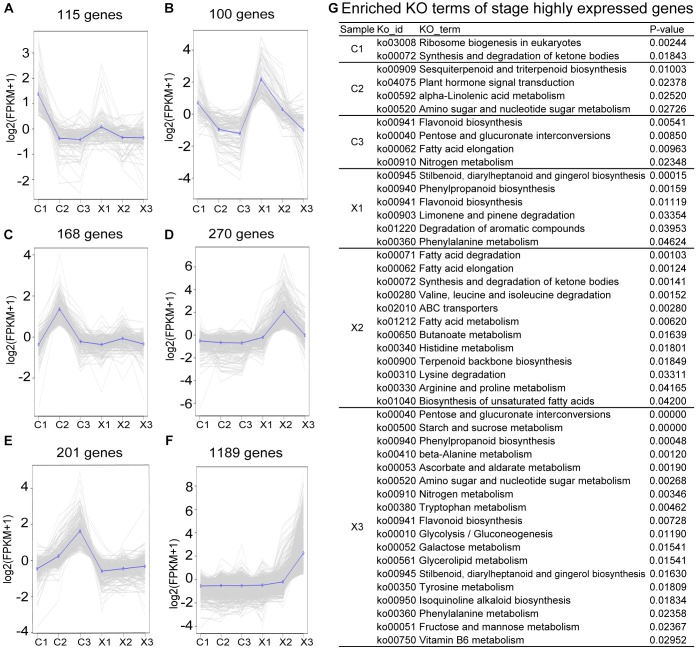
Analysis of stage-specific genes in male and female flowers. **(A–F)** Expression patterns of specific genes to each developmental stage of female flowers, with the average expression trend highlighted. **(G)** Enriched KO terms among stage-specific genes (*p*-values ≤ 0.05).

In stage 2 (C1, X1), a total of 115 genes were specifically expressed in C1 that were enriched for ribosome biogenesis in eukaryotes (ko03008) and synthesis and degradation of ketone bodies (ko00072) by KEGG enrichment analysis (Figure 3A,G and Supplementary Table S3), including *Early flower 4* (*ELF4*) gene and *ELF4-LIKE 4* (*EFL4*) gene involved in the flowering time and embryoid’s morphogenesis of *Arabidopsis*, and male fertility genes, like *G-type lectin S-receptor-like serine/threonine-protein kinase RLK1, Non-specific lipid-transfer protein 8*, and *Myb family transcription factor At1g14600* (Boutrot et al., 2006; Xiang et al., 2007; Wan et al., 2008; Adeyemo et al., 2011). A total of 100 stage-specific genes in X1 were enriched for stilbenoid, diarylheptanoid and gingerol biosynthesis (ko00945), phenylpropanoid biosynthesis (ko00940), flavonoid biosynthesis (ko00941), limonene and pinene degradation (ko00903), degradation of aromatic compounds (ko01220), and phenylalanine metabolism (ko00360), including *Leucine-rich repeat receptor protein kinase EXS*, *G-type lectin S-receptor-like serine/threonine-protein kinase RLK1*, *Phenylalanine ammonia-lyase (PAL)*, *Argonaute 2* (*AGO2*). These genes are well-known to function in anther or pollen development (Zhao et al., 2002; Xiang et al., 2007; Wan et al., 2008; Feng et al., 2017) (Figure 3B and Supplementary Table S3).

In stage 4 (C2, X2), SFF began to degenerate in C2, while SMF developed at microspore stage. KEGG enrichment analysis showed that a total of 168 stage-specific genes were mainly enriched in sesquiterpenoid and triterpenoid biosynthesis (ko00909), plant hormone signal transduction (ko04075), alpha-Linolenic acid metabolism (ko00592), Amino sugar and nucleotide sugar metabolism (ko00520), including *Vacuolar cation/proton exchanger 3* (*CAX3*), *Arabinogalactan peptide 16* (*AGP16*), *Calcium-binding protein CML 38* (*CML38*), *HVA22-like protein A* (*HVA22A*), and *E3 ubiquitin-protein ligase PUB 23* (*PUB23*). These genes have been reported to function in PCD (Guo and Ho, 2008; Orosa et al., 2017; Sala et al., 2017) (Figure 3C,G and Supplementary Table S3). Moreover, the C2-specific genes included a number of transcription factors such as *WRKY*s (*WRKY18*, *WRKY46*), *bHLH*s (*bHLH96*, *bHLH3*), *MYB* (*MYB108*), etc. (Supplementary Table S3), indicating that the transcription factors should play a role in the abortion of SFF. However, there were 270 specific genes in X2 mainly enriched in 12 pathways, including fatty acid degradation (ko00071), fatty acid elongation (ko00062), synthesis and degradation of ketone bodies (ko00072), etc. (Figure 3D,G and Supplementary Table S3).

In stage 6 (C3, X3), a total of 201 specific genes were identified in C3, while 1189 specific genes were in X3 (Figure 3E,F and Supplementary Table S3). KEGG enrichment analysis showed that the C3-specific genes were enriched in flavonoid biosynthesis (ko00941), pentose and glucuronate interconversions (ko00040), fatty acid elongation (ko00062), nitrogen metabolism (ko00910) (Figure 3G). Besides, PCD-related genes including *HVA22-like C* (*HVA22C*) and *Protein ASPARTIC PROTEASE IN GUARD CELL* (*ASPG*), ovule development controlling gene, *Agamous-like 11* (*AGL11*), and petal development controlling gene, *AGL32* were also included in C3 (Supplementary Table S3). In X3, the 1189 specific genes were mainly enriched in 18 pathways, including pentose and glucuronate interconversions (ko00040), starch and sucrose metabolism (ko00500), and phenylpropanoid biosynthesis (ko00940), etc. (Figure 3G).

### Identification of Candidate Genes Involved in Anther and Pollen Development in the Abortion of SFF

**FIGURE 4:**
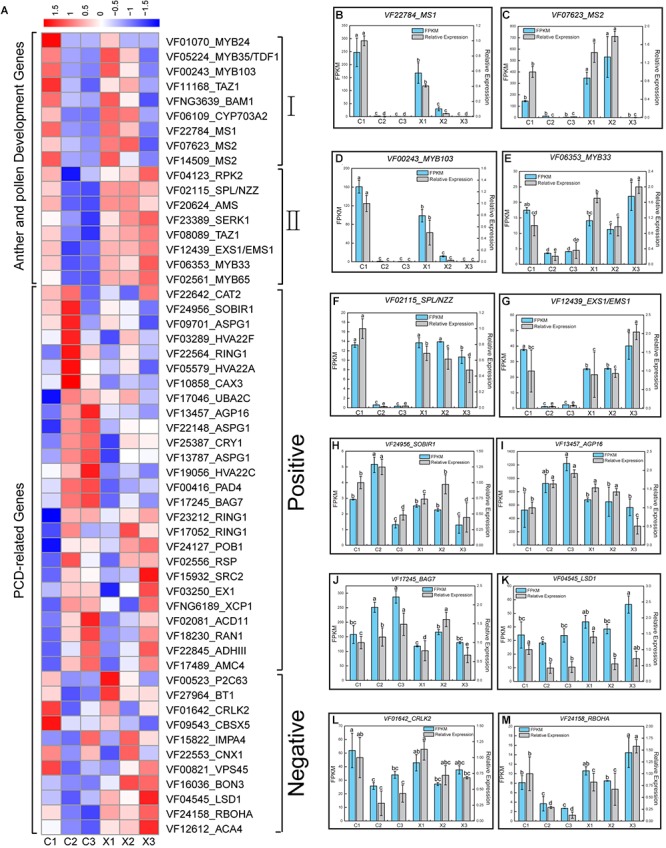
Expression patterns of anther and pollen development genes and PCD-related genes in male and female flowers. **(A)** Heatmap analysis of the important anther and pollen development genes and PCD-related genes in male and female flowers; I, Genes highly expressed in C1, X1, and X2; II, Genes highly express in C1, X1, X2, and X3. **(B–G)** Expression levels of *MS1*, *MS2*, *MYB103*, *MYB33*, *SPL/NZZ*, and *EXS1/EMS1* based on FPKM and qRT-PCR data. **(H–M)** Expression levels of *SORB1*, *AGP16*, *BAG7*, *LSD1*, *SRLK2*, and *ROHBA* based on FPKM and qRT-PCR data. Error bars indicate SD. Different letters represent significant difference in mRNA levels (*p* < 0.05).

Cytological observation confirmed that the tapetum cell degeneration may lead to the abortion of SFF in tung tree. A total of 17 key genes were reported to function in anthers and pollens, and they showed two different expression patterns in tung flowers. Pattern 1 included nine genes *MYB-related protein Zm38* (*MYB35/TDF1*), *MYB24*, *ODORANT 1* (*MYB103/ODO1*), *BTB/POZ and TAZ domain-containing protein 3* (*TAZ1*), *BARELY ANY MERISTEM 1* (*BAM1*), *Cytochrome P450 703A2* (*CYP703A2*), *MALE STERILITY* (*MS1*), and *Fatty acyl-CoA reductase 2* (*MS2*) (Zhu et al., 2010; Huang et al., 2017) which were highly expressed in C1, X1, and X2 stages (Figure 4A and Supplementary Table S4). Pattern 2 included eight genes *LRR receptor-like serine/threonine-protein kinase RPK 2* (*RPK2*), *SPOROCYTELESS/NOZZLE* (*SPL/NZZ*), *ABORTED MICROSPORES* (*AMS*) *Somatic embryogenesis receptor-like kinase 1* (*SERK1*), *TAZ1*, *EXCESS MICROSPOROCYTES1/EXTRA SPOROGENOUS CELLS* (*EXS1/EMS1*), *MYB33* and *MYB65* (Millar and Gubler, 2005; Ferguson et al., 2017; Guo et al., 2018), which were highly expressed in C1, X1, X2, and X3 stages (Figure 4A and Supplementary Table S4). QRT-PCR was applied to validate the expression of six genes of anther and pollen at different developmental stages of male and female flowers and generated consistent expression patterns with RNA-Seq data (Figure 4B–G). Together, these 17 key genes in anther and pollen development were suppressed in C2 and C3, and their low expression or silence might result in tapetum and microspore degeneration in tung tree.

### Identification of Candidate Genes Involved in PCD in the Abortion of SFF

Cytological observation confirmed that the abortion of SFF belongs to PCD type in tung tree. To gain insights into the role of PCD-related genes in the abortion of SFF, the putative 37 key PCD-related genes were identified based on their expression patterns which included 26 positive genes and 11 negative genes (Figure 4A and Supplementary Table S5). Most of the positive genes exhibited high expression levels in C2 and C3, such as *Leucine-rich repeat receptor-like serine/threonine/tyrosine-protein kinase* (*SOBIR1*), *AGP16*, and *BAG family molecular chaperone regulator 7* (*BAG7*) etc. (Figure 4A and Supplementary Table S5). These genes have been reported to behavior in PCD (Gao and Showalter, 1999; Li et al., 2017; Sala et al., 2017). Likewise, negative genes showed low expression levels in C2 and C3 and high expression in C1 such as *Lesion Simulating Disease 1 (LSD1*), *Calcium/calmodulin-regulated receptor-like kinase 2* (*CRKL2*), *Homologies of Respiratory Burst oxidase homolog protein A* (*RBOHA*) and so on (Torres et al., 2002; Yang et al., 2010; Guo et al., 2013) (Figure 4A and Supplementary Table S5). The qRT-PCR analysis of six genes revealed consistent expression patterns with those generated by RNA-seq data (Figure 4H–M). The expression patterns demonstrated the important role of PCD-related genes in the abortion of SFF in tung tree.

### Phytohormone Levels in Male and Female Flowers

To explore the possible phytohormone regulation in the abortion of SFF, the endogenous levels of auxin, abscisic acid (ABA), gibberellin (GA), jasmonic acid (JA), cytokinin (CK), and SA in tung flowers at different stages were measured (Table 1). The male flower exhibited the same patterns of IAA, ABA, and GA levels with the female flower. The IAA level was up-regulated reaching the highest at stage 3, and it was significantly higher in X3 (8.35 ng/g) than that in C3 (1.68 ng/g) (Table 1). The ABA levels were down-regulated across three stages, and it was significantly higher in C1 (165.68 ng/g) than that in X1 (87.64 ng/g) (Table 1). For GAs, GA4 level was significantly higher than GA1, GA3, and GA7 in all samples. In addition, the GA4 level was the lowest in C2 (1.66 ng/g) and X2 (1.0 ng/g) (Table 1).

**Table 1:** Levels of phytohormones in male and female flowers of tung tree.

Hormone types		Female flowers stage			Male flowers stage		
		C1	C2	C3	X1	X2	X3
IAA	IAA	0.23 ± 0.05 e	0.53 ± 0.04 d	1.68 ± 0.06 b	0.11 ± 0.01 e	1.29 ± 0.07 c	8.35 ± 0.29 a
ABA	ABA	165.68 ± 1.17 a	80.37 ± 1.05 c	15.01 ± 0.28 e	87.64 ± 0.57 b	45.42 ± 1.26 d	15.73 ± 0.81 e
GAs	GA_1_	ND	ND	0.23 ± 0.03 c	ND	0.61 ± 0.02 b	0.44 ± 0.06 a
	GA_3_	0.06 ± 0 a	0.02 ± 0.01 c	0.01 ± 0 c	0.01 ± 0 c	0.04 ± 0 b	0.06 ± 0.01 a
	GA_4_	3.65 ± 0.1 c	1.66 ± 0.11 d	4.24 ± 0.14 b	1.44 ± 0.08 d	1 ± 0 e	11.64 ± 0.16 a
	GA_7_	0.3 ± 0.04 a	0.02 ± 0 b	0.02 ± 0 b	0 ± 0 b	ND	0 ± 0 b
CKs	ZT	ND	0.06 ± 0.01 bc	0.06 ± 0.01 bc	0.04 ± 0 bc	0.1 ± 0.02 b	0.48 ± 0.11 a
	TZR	6.33 ± 0.32 d	7.16 ± 0.3 c	7.46 ± 0.22 c	1.72 ± 0.02 e	11.29 ± 0.25 b	52.45 ± 0.52 a
	iP	0.13 ± 0.03 b	0.03 ± 0 c	0.05 ± 0 c	1.11 ± 0.07 a	0.06 ± 0.01 c	0.04 ± 0.01 c
	iPA	0.63 ± 0.03 e	0.56 ± 0.04 e	0.93 ± 0.04 d	3.15 ± 0.16 a	1.97 ± 0.18 b	1.3 ± 0.21 c
SAs	SA	ND	16.93 ± 0.18 a	0.88 ± 0.24 b	ND	ND	0.86 ± 0.05 b
	MESA	0.04 ± 0 a	0.01 ± 0 b	ND	ND	ND	ND
JAs	JA	7.04 ± 0.07 d	8.93 ± 0.19 c	7.37 ± 0.2 d	5.54 ± 0.02 e	10.35 ± 0.29 b	129.4 ± 0.44 a
	MEJA	0.14 ± 0.02 c	0.56 ± 0.05 b	0.17 ± 0.01 c	0.14 ± 0.01 c	0.27 ± 0.03 c	2.24 ± 0.2 a


*Endogenous phytohormone levels were measured in triplicate and the mean values ± SD (ng.g^-*1*^) are shown in each sample. Different letters in each row represent significantly differences between samples at 5% level. ND, not detected. IAA, indole-3-acetic acid; ABA, abscisic acid; GAs, gibberellins; CK, cytokinin; ZT, zeatin; TZR, *trans-*Zeatin-riboside; iP, *N*^*6*^-isopentenyl adenine; iPA, *N*^*6*^-isopentenyl adenosine; SA, salicylic acid; MESA, methyl salicylate; JA, jasmonic acid; MEJA, methyl jasmonate*.

The levels of JAs and CKs showed different patterns in comparison with IAA, ABA, and GA (Table 1). JA was significantly higher than MEJA across all samples. It was up-regulated in male flowers with the highest value (129.4 ng/g) in X3 (Table 1). For CKs, the TZR level was significantly higher than Zeatin, IP and IPA. The TZR level was up-regulated in both male and female flowers (Table 1). Notably, SA showed different patterns from other phytohormones. No SA was detected in C1, X1 and X2, and only extremely low levels of SA were detected in C3 (0.88 ng/g) and X3 (0.86 ng/g) (Table 1). In contrast, SA was significantly high in C2 (16.93 ng/g) where the abortion of SFF occurred.

According to the analysis of phytohormone levels, SA is likely to play an important role in the abortion of SFF instead of other phytohormones.

### Analysis of SA Synthesis and Signaling Pathway

**FIGURE 5:**
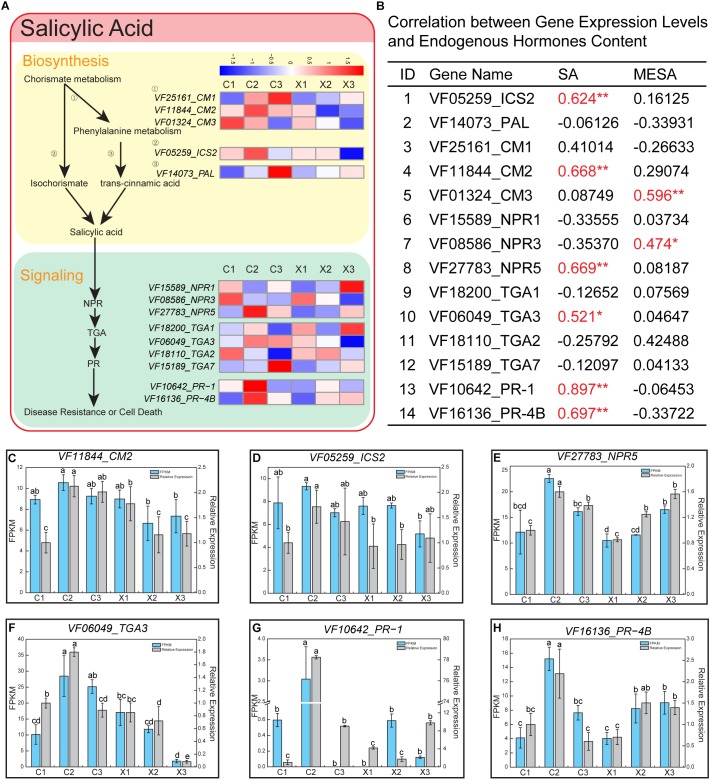
Salicylic acid (SA) pathway in male and female flowers. **(A)** Representative gene expression profiles of SA biosynthesis and signaling pathway. The color intensity in the heatmap of each gene represents the relative expression levels of these genes at different stages of male and female flowers. **(B)** Correlation between gene expression levels and endogenous hormones content in male and female flowers. “^∗^” and “^∗∗^” after the correlation coefficients represent the correlation between their mRNA levels and endogenous hormone levels significantly different at *p* < 0.05 and *p* < 0.01, respectively. **(C–H)** Expression levels of *CM2*, *ICS2*, *NPR5*, *TGA3*, *PR-1*, and *PR-4B* based on FPKM and qRT-PCR data. Error bars indicate SD. Different letters represent significant difference in mRNA levels (*p* < 0.05).

To uncover the regulatory mechanism of SA involved in the abortion of SFF, we analyzed in detail the pathway of SA synthesis and signaling in tung flowers. Two pathways have been reported to be involved in plant SA synthesis (Chen et al., 2009). One is regulated by phenylalanine ammonia lyase (PAL) and chorismate mutase (CM), and the other by isochorismate synthase (ICS). In *Arabidopsis*, there are four homologs encoding *PAL* (*PAL1-4*), three homologs encoding *CM* (*CM1-3*), and two homologs encoding *ICS* (*ICS1/2*) (Huang et al., 2010). In tung tree genome, we found three *CM* members (*CM1-3*), one *PAL* and one *ICS* (*ICS2*). The three *CM*s displayed different expression patterns. Generally, FPKM of *CM1* was higher than *CM2* and *CM3* across all samples. *CM1* displayed different expression profiles in male and female flowers, and was up-regulated with the development of female flowers (Figure 5A). *CM2* was highly expressed in C2 of female flowers, and down-regulated in male flowers (Figure 5A and Supplementary Table S6). *CM3* was down-regulated in both male and female flowers (Figure 5A and Supplementary Table S6). *PAL* was up-regulated in both male and female flowers. *ICS2* was highly expressed in C2 of female flowers, and down-regulated in male flowers (Figure 5A and Supplementary Table S6).

In *Arabidopsis* SA signaling pathway, three receptors are reported, including NON-EXPRESSOR OF PR1 (NPR), transcription factor TGA (TGA), PATHOGENESIS-RELATED (PR) (Kaltdorf and Naseem, 2013). Three homologs of *NPR* (*NPR1/3/5*), four *TGA*s (*TGA1-3*, *TGA7*), and two *PR*s (*PR-1*, *PR-4B*) were found in tung tree genome (Figure 5A and Supplementary Table S6). The expression level of *NPR3* was higher than *NPR1* and *NPR5*, and down-regulated in male and female flowers of tung tree. *NPR1* was down-regulated in female flowers, but up-regulated in male flowers. *NPR5* was highly expressed in C2 of female flowers, and up-regulated in male flowers (Figure 5A and Supplementary Table S6). Among the TGA family, *TGA3* showed higher expression level than the other three members. It was highly expressed in C2 of female flowers, and down-regulated in male flowers (Figure 5A and Supplementary Table S6). Interestingly, two members of the PR family (*PR-1*, *PR-4B*) were highly expressed in C2 of female flowers (Figure 5A and Supplementary Table S6).

Correlation analysis revealed that expression levels of six genes among the selected 14 genes were highly correlated with SA, including *ICS2*, *CM2*, *NPR5*, *TGA3*, *PR-1*, and *PR-4B*, while only two genes (*CM3* and *NPR3*) were significantly correlated with MESA (Figure 5B). Besides, the qRT-PCR analysis of six genes revealed consistent expression patterns with those generated by RNA-seq data (Figure 5C–H). Based on the above analyses, *ICS2*, *CM2*, *NPR5*, *TGA3*, *PR-1*, and *PR-4B* should be the important regulators in the pathways of SA synthesis and signaling and play a role in the abortion of SFF in tung tree.

### Co-expression Networks Analysis of Expression Genes Involved in the Abortion of SFF

**FIGURE 6:**
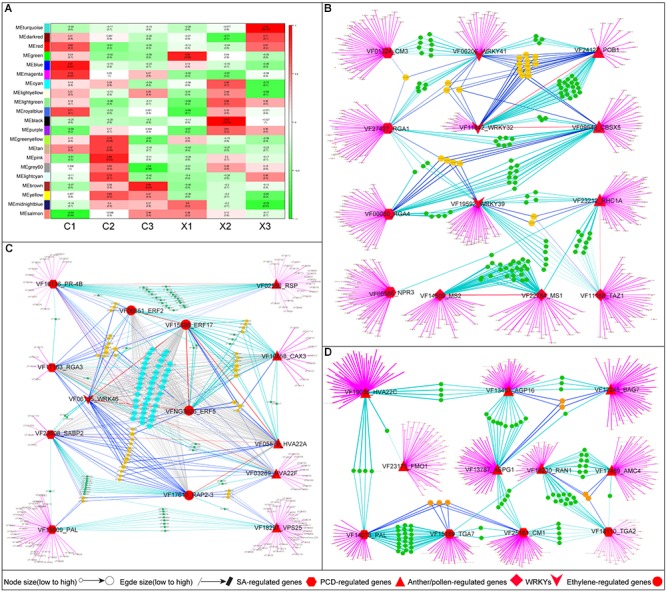
Co-expression networks analysis of genes in different module of female flowers in tung tree. **(A)** Module trait relationships in six samples. **(B–D)** Co-expression networks analysis of anther and pollen development genes, SA- and PCD-related genes in MEblue, MEpink, and MEbrown module.

To gain more insights into the regulatory relationships of anther and pollen development genes, SA- and PCD-related genes in female flowers of tung tree, we performed a WGCNA of transcript expression in male and female flowers (FPKM values ≥ 1). Consequently, 21 co-expression modules were identified for each sample (Figure 6A). An effective PCC threshold of ≥ 0.85 and a *p*-value of ≤ 0.05 were trained to generate the significant modules. Finally, we found that each sample of female flowers had one significant module, namely C1 with MEblue module (PCC = 0.91, *p*-value = 0.01), C2 with MEpink module (PCC = 0.88, *p*-value = 0.02), and C3 with MEbrown module (PCC = 0.85, *p*-value = 0.03) (Figure 6A).

The MEblue module of the co-expression network, representing the development of SFF, contained 3469 genes. Of the 3469 genes, a number of genes were identified as anther and pollen development genes (*TAZ1*, *MS1*, *MS2*), PCD-related genes (*RING1*, *CBSX5*, and *POB1*), and SA-related genes (*CM3*, *NPR3*, *RGA1*, *RGA4*) (Supplementary Table S7). The *TAZ1* (2324 edges), *MS1* (2583), *MS2* (2297), *RING1* (2926), *POB1* (3159), *CM3* (2512), *NPR3* (2534), *RGA1* (2990), and *RGA4* (3132) had significantly high edge numbers, suggesting their prominent roles in anther and pollen development of female flowers (Figure 6B and Supplementary Table S7). Furthermore, *WRKY*s were identified as having high connectivity between SA- and PCD-related genes, or SA-related genes and anther and pollen development genes, i.e., *WRKY41* (3132 edges), *WRKY32* (3178), *WRKY39* (2992), suggesting their important biological functions in regulating anther and pollen development of female flowers in tung tree (Figure 6B and Supplementary Table S7).

The MEpink module represented the beginning of the SFF abortion contained 533 genes in which many PCD-, SA- and ethylene-related genes showed high connectivity in the network (Supplementary Table S7), such as (1) the PCD-related genes of *VPS25* (354 edges), *CAX3* (326), *HVA22A* (373), and *HVA22F* (375); (2) the SA-related genes of *PAL* (408), *PR-4B* (435), and *SABP2* (375), (3) the ethylene-related genes of ethylene-responsive transcription factors (*ERF2* (378), *ERF5* (379), *ERF17* (373), and *RAP2-3* (409)) (Figure 6C and Supplementary Table S7). Interestingly, anther and pollen development genes were not found in MEpink module. Furthermore, only one *WRKY* (*WRKY46*, 434 edges) was identified as having high connectivity in MEpink module (Figure 6C and Supplementary Table S7). Based on these findings, PCD, SA and ethylene may have critical roles in regulating the abortion of SFF in tung tree.

The MEbrown module, contained 2453 genes and represented the abortion of SFF in tung tree (Supplementary Table S7). Interestingly, only PCD- and SA-related genes were detected in the network, such as *AGP16*, *RAN1*, *BAG7, FLAVIN-DEPENDENT MONOXYGENASE1* (*FMO1*), *ASGP1*, *HVA22C*, *CM1*, *TGA7*, and *PAL, etc.* (Supplementary Table S7). Furthermore, there were high connectivity among (1) the PCD-related genes, including *AGP16* (1872 edges), *RAN1* (1509), *BAG7* (1925), and *FMO1* (1010), (2) the SA-related genes, including *CM1* (2114), and *PAL* (1660) (Figure 6D and Supplementary Table S7). This result suggests that these genes play a key roles in regulating the abortion of SFF in tung tree.

Taken together, the analyses of gene co-expression networks suggested that SA might regulate the anther and pollen development and the PCD, resulting in the abortion of SFF in tung tree.

## Discussion

The production of unisexual flowers in flowering plants has evolved more than 1000 times (Renner and Feil, 1993). Unisexual flowering systems promote outbreeding and are considered as driving forces in plant evolution. Although unisexual flowering systems have been studied in *A. deliciosa*, *Quercus sube*, and *Diospyros kaki* (Rocheta et al., 2014; Akagi et al., 2016, 2018), the regulatory mechanisms are unclear.

### Anther and Pollen Development Genes Regulate the Abortion of SFF

In tung tree, we found that the SFF was arrested in C2 before the formation of tetrads in microspore meiosis (Figure 1), which was similar with *Asparagus officinalis* (Caporali et al., 1994). More importantly, the abortion of SFF began with tapetum degeneration. Throughout the abortion process of SFF, the degeneration of tapetum may be a key factor causing the MMC degeneration in SFF. The tapetum plays an important role in pollen grain development which serves as a nutritive tissue, providing metabolites, nutrients, and cell wall precursors for the development of pollen grains, therefore any obstruction of the tapetum development will lead to male sterility in plants (Pacini, 1997; Chen et al., 2018). In tung tree, the tapetum degeneration may result in the abortion of SFF.

The use of classical genetic screens has uncovered a large number of genes involved in anthers development and in the production of viable pollen. In our study, 17 anther and pollen development genes were highly expressed in C1, but lowly expressed or silenced in C2 and C3 (Figure 4). It is worth mentioning that *SPL/NZZ*, and *EXS1/EMS1* have important roles in early anther development and differentiation, while *AMS*, *MS1*, *MYB33*, *MYB65*, and *MYB103* are involved in tapetum and pollen wall development (Pearce et al., 2015). Thus, low expression or silence of anther and pollen development genes, especially the genes of tapetum development, may result in the abortion of SFF in C2 and C3 of female flowers.

### PCD Triggers the Abortion of SFF

Programmed cell death is a controlled cellular suicide which plays an important role in plant development. The nuclear DNA degradation, vacuolization, or the activation of specific proteases belongs to specific morphological and biochemical features of PCD (Hautegem et al., 2015). In tung tree, SFF were degenerated when stamens developed into early MMC, and MMC were vacuolize in C2 of female flowers (Figure 1). In *Thymelaea* and *Meliaceae*, the gynoecium in male flowers is aborted by PCD at meiosis stage (Caporali et al., 2006; Gouvea et al., 2008). Hence, PCD is essential to many aspects of plant morphogenesis and is a normal component of flower development (Rogers, 2006).

Programmed cell death is controlled by both positive regulators and negative regulators. In this study, positive regulators were highly expressed in C2 or C3 of female flowers, like *SOBIR1*, *ASPG16*, and *BAG7*, which are well-known to positively regulate PCD (Gao and Showalter, 1999; Li et al., 2017; Lu et al., 2017; Sala et al., 2017). Negative regulators showed low expression in C2 and C3, such as *RBOH*s, which are the source of reactive oxygen species in the oxidative burst and negatively regulate cell death (Torres et al., 2002). These results suggested that PCD-related genes rapidly caused the abortion of SFF in tung tree.

### Plant Hormones Are Associated With the Abortion of SFF

A variety of plant hormones are likely to be involved in the regulation of reproductive organ abortion and development pathways in unisexual flowers (Ubeda-Tomas and Bennett, 2010). For example, ethylene increases the percentage of female flowers in *Cucumis sativus* and *Cucumis melo* (Boualem et al., 2008; Tao et al., 2018). CK promotes female flowers development in *Populus tomentosa*, *Jatropha curcas*, and *Castanea henryi* (Song et al., 2013; Chen et al., 2014; Fan et al., 2017). In tung tree, contents of GA4, IAA, TZR and JA were significantly higher in X3 of male flowers than C3 of female flowers (Table 1). Only SA was specifically and highly expressed in C2, the initial stage of the abortion of SFF (Table 1). Therefore, the SA accumulation may suppress development of SFF in tung tree.

*NPR5*, *TAG3*, *PR-1*, and *PR-4B* in SA pathway showed high expression in C2 of female flowers. Interestingly, *ICS2* also exhibited the highest expression level in C2 among all samples. In *Arabidopsis*, isochorismate pathway is the main source of SA accumulation (Wildermuth et al., 2001). Further, the isochorismate pathway also has been shown to be active in tomato and tobacco (Uppalapati et al., 2007). Together, isochorismate pathway may be also the main source of large SA accumulation in C2 of female flowers in tung tree.

### SA Mediates the Abortion of SFF by Anther and Pollen Development Genes and PCD-Related Genes

Salicylic acid mediates PCD in plants under biotic stress (Alvarez, 2000). In our study, co-expression networks analysis indicated that anther and pollen development genes, SA- and PCD-related genes showed high connectivity in the network, and that *WRKY*s were the important connectors between SA-related genes and anther and pollen development genes, and SA- and PCD-related genes (Figure 6). In *Arabidopsis*, members of *WRKY*s have interaction with SA-related genes. For example, *WRKY58* interacts with *NPR1* to regulate SA accumulation in plant defense (Wang et al., 2006). The lower level of SA limits *NPR1*-*NPR3* interaction, enabling *NPR1* to restrict the spread of PCD (Fu et al., 2012). SA-dependent signal potentiation loop has been proposed to be negatively regulated by protein LSD1 (Aviv et al., 2002), which also explains the runaway cell death phenotype of the *lsd1* mutant. In addition, FMO1 is not only necessary for systemic accumulation of SA and downstream signaling after pathogen infection but also regulates PCD in *Arabidopsis* (Xu and Brosche, 2014). In rice, SA is suggested to regulate pollen viability and floret fertility (Zhao et al., 2018). Herein, we proposed a possible signaling network where anther and pollen development genes and PCD-related genes work synergistically with SA-related genes to induce the abortion of SFF in tung tree (Figure 6).

**FIGURE 7:**
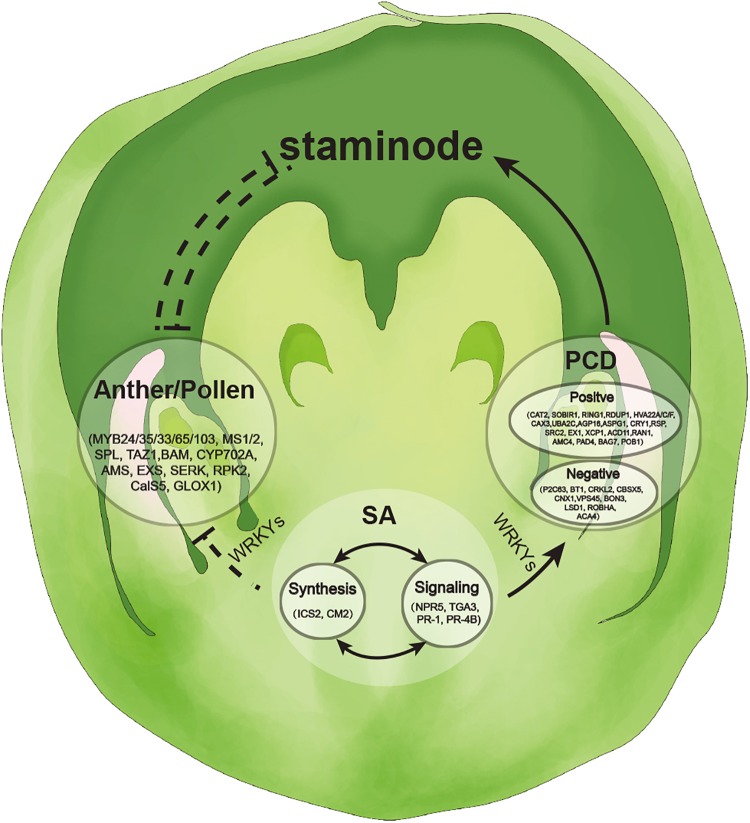
Hypothetical model for development of female flowers in tung tree.

Additionally, we constructed a hypothetical model for the development of normal female flowers in tung tree. At the early stage, SFF develops normally in female flowers with low level of SA (Figure 7). With SA accumulation, PCD-related genes are activated and highly expressed, and subsequently anther and pollen development genes, especially tapetum genes are suppressed (Figure 7). Consequently, the abortion of SFF occurs and the normal female flower develops.

## Conclusion

We confirmed that the abortion of SFF in tung tree belongs to the PCD type and demonstrated that tapetum degeneration at MMC stage is the major reason causing the abortion of SFF. Furthermore, we constructed a model for the abortion of SFF in tung tree based on integrated analyses of morphological and cytological observations, endogenous phytohormone assay and RNA-seq. During the middle stage in female flowers, SA accumulation triggers PCD activation and arrests anther and pollen development, which ultimately results in the abortion of SFF in tung tree. This study provides valuable information for better understanding the development of female flowers in tung tree.

## Author Contributions

LZ, XT, and ML conceived and designed the experiments. ML, WL, XF, and GZ performed the experiments. ML, XF, HL, MS, GZ, YF, and LZ analyzed the data. ML, WL, GZ, XT, HL, YF, and LZ contributed reagents, materials, and analysis tools. ML and LZ wrote the manuscript. All authors discussed results and commented on the manuscript.

## Conflict of Interest Statement

The authors declare that the research was conducted in the absence of any commercial or financial relationships that could be construed as a potential conflict of interest.

## Footnotes

1 http://www.cytoscape.org/

## Supplementary Material

FIGURE S1 Tung tree flower and fruit. **(A,D)** Male flower. **(B,E)** Female flower. **(C,F)** Bisexual flower. **(G,H)** Tung fruit of female and bisexual flowers. **(I)** Fruiting ratio of female and bisexual flowers.

TABLE S1 Primer of qRT-PCR.

TABLE S2 KEGG enrichment analysis of DEGs in male and female flowers.

TABLE S3 Stage-specific genes analysis in male and female flowers.

TABLE S4 Expression patterns of anther and pollen development genes in male and female flowers.

TABLE S5 Expression patterns of PCD-related genes in male and female flowers.

TABLE S6 Salicylic acid pathway in male and female flowers.

TABLE S7 Co-expression networks analysis of genes in different module of female flowers in Tung Tree.

## References

[B1] Adeyemo O. S. Kolmos E. Tohme J. Chavariaga P. Fregene M. Davis S. J. (2011). Identification and characterization of the cassava core-clock gene early flowering 4. *Trop. Plant Biol.* 4 117–125. 10.1007/s12042-011-9065-6

[B2] Akagi T. Henry I. M. Kawai T. Comai L. Tao R. (2016). Epigenetic regulation of the sex determination gene MeGI in polyploid persimmon. *Plant Cell* 28 2905–2915. 10.1105/tpc.16.00532 27956470 PMC5240738

[B3] Akagi T. Henry I. M. Ohtani H. Morimoto T. Beppu K. Kataoka I. (2018). A Y-encoded suppressor of feminization arose via lineage-specific duplication of a cytokinin response regulator in *Kiwifruit*. *Plant Cell* 30 780–795. 10.1105/tpc.17.00787 29626069 PMC5969274

[B4] Alvarez M. E. (2000). Salicylic acid in the machinery of hypersensitive cell death and disease resistance. *Plant Mol. Biol.* 44 429–442. 10.1023/A:1026561029533 11199399

[B5] Anders S. Huber W. (2010). Differential expression analysis for sequence count data. *Genome Biol.* 11:R106. 10.1186/gb-2010-11-10-r106 20979621 PMC3218662

[B6] Arrom L. Munne-Bosch S. (2012). Sucrose accelerates flower opening and delays senescence through a hormonal effect in cut lily flowers. *Plant Sci.* 188 41–47. 10.1016/j.plantsci.2012.02.012 22525243

[B7] Aviv D. Rustérucci C. Holt B. Dietrich R. Parker J. Dangl J. (2002). Runaway cell death, but not basal disease resistance, in lsd1 is SA- and NIM1/NPR1-dependent. *Plant J.* 29 381–391. 10.1046/j.0960-7412.2001.01225.x 11844114

[B8] Barrett S. C. H. (2010). Understanding plant reproductive diversity. *Philos. T. R. Soc. B.* 365 99–109. 10.1098/rstb.2009.0199 20008389 PMC2842705

[B9] Boualem A. Fergany M. Fernandez R. Troadec C. Martin A. Morin H. (2008). A conserved mutation in an ethylene biosynthesis enzyme leads to andromonoecy in melons. *Science* 321 836–838. 10.1126/science.1159023 18687965

[B10] Boutrot F. Meynard D. Guiderdoni E. Joudrier P. Gautier M. F. (2006). The *Triticum aestivum* non-specific lipid transfer protein (TaLtp) gene family: comparative promoter activity of six TaLtp genes in transgenic rice. *Planta* 225 843–862. 10.1007/s00425-006-0397-7 16983534

[B11] Cahoon E. B. Carlson T. J. Ripp K. G. Schweiger B. J. Cook G. A. Hall S. E. (1999). Biosynthetic origin of conjugated double bonds: production of fatty acid components of high-value drying oils in transgenic soybean embryos. *Proc. Natl. Acad. Sci. U.S.A.* 96 12935–12940. 10.1073/pnas.96.22.12935 10536026 PMC23170

[B12] Caporali E. Carboni A. Galli M. G. Rossi G. Spada A. Longo G. P. M. (1994). Development of male and female flower in Asparagus officinalis. Search for point of transition from hermaphroditic to unisexual developmental pathway. *Sex. Plant Reprod.* 7 239–249. 10.1007/BF00232743

[B13] Caporali E. Roccotiello E. Cornara L. Casazza G. Minuto L. (2006). An anatomical study of floral variation in *Thymelaea hirsuta*(L.) Endl. related to sexual dimorphism. *Plant Biosyst.* 140 123–131. 10.1080/11263500600756199

[B14] Chen M. S. Pan B. Z. Wang G. J. Ni J. Niu L. Xu Z. F. (2014). Analysis of the transcriptional responses in inflorescence buds of *Jatropha curcas* exposed to cytokinin treatment. *BMC Plant Biol.* 14:318. 10.1186/s12870-014-0318-z 25433671 PMC4272566

[B15] Chen Y. H. Chen J. H. Chang C. Y. Chang C. C. (2010). Biodiesel production from tung (*Vernicia montana*) oil and its blending properties in different fatty acid compositions. *Biores. Technol.* 101 9521–9526. 10.1016/j.biortech.2010.06.117 20702090

[B16] Chen Z. Zheng Z. Huang J. Lai Z. Fan B. (2009). Biosynthesis of salicylic acid in plants. *Plant Signal Behav.* 4 493–496. 10.4161/psb.4.6.8392 19816125 PMC2688294

[B17] Chen Z. S. Liu X. F. Wang D. H. Chen R. Zhang X. L. Xu Z. H. (2018). Transcription factor OsTGA10 is a target of the MADS protein OsMADS8 and is required for Tapetum development. *Plant Physiol.* 176 819–835. 10.1104/pp.17.01419 29158333 PMC5761795

[B18] Cheng P. C. Greyson R. I. Walden D. B. (1983). Organ initiation and the development of unisexual flowers in the tassel and ear of *zea mays*. *Am. J. Bot.* 70 450–462. 10.2307/2443252

[B19] Coimbra S. Torrao L. Abreu I. (2004). Programmed cell death induces male sterility in *Actinidia deliciosa* female flowers. *Plant Physiol. Biochem.* 42 537–541. 10.1016/j.plaphy.2004.05.004 15246067

[B20] Cui P. Lin Q. Fang D. Zhang L. Li R. Cheng J. (2018). Tung tree (*Vernicia fordii*) genome and transcriptome sequencing reveals coordinate upregulation of fatty acid beta-oxidation and triacylglycerol biosynthesis pathways during eleostearic acid accumulation in seeds. *Plant Cell Physiol.* 59 1990–2003. 10.1093/pcp/pcy117 30137600

[B21] Fan X. M. Yuan D. Y. Tian X. M. Zhu Z. J. Liu M. L. Cao H. P. (2017). Comprehensive transcriptome analysis of phytohormone biosynthesis and signaling genes in the flowers of chinese chinquapin (*Castanea henryi*). *J. Agri. Food Chem.* 65 10332–10349. 10.1021/acs.jafc.7b03755 29111713

[B22] Feng N. Song G. Guan J. Chen K. Jia M. Huang D. (2017). Transcriptome profiling of wheat inflorescence development from spikelet initiation to floral patterning identified stage-specific regulatory genes. *Plant Physiol.* 174 1779–1794. 10.1104/pp.17.00310 28515146 PMC5490901

[B23] Ferguson A. C. Pearce S. Band L. R. Yang C. Y. Ferjentsikova I. King J. (2017). Biphasic regulation of the transcription factor ABORTED MICROSPORES (AMS) is essential for tapetum and pollen development in *Arabidopsis*. *New Phytol.* 213 778–790. 10.1111/nph.14200 27787905 PMC5215365

[B24] Florea L. Song L. Salzberg S. L. (2013). Thousands of exon skipping events differentiate among splicing patterns in sixteen human tissues. *F1000Res.* 2:188. 10.12688/f1000research.2-188.v2 24555089 PMC3892928

[B25] Fu Z. Q. Yan S. P. Saleh A. Wang W. Ruble J. Oka N. (2012). NPR3 and NPR4 are receptors for the immune signal salicylic acid in plants. *Nature* 486 228–232. 10.1038/nature11162 22699612 PMC3376392

[B26] Gao M. Showalter A. M. (1999). Yariv reagent treatment induces programmed cell death in *Arabidopsis* cell cultures and implicates arabinogalactan protein involvement. *Plant J.* 19 321–331. 10.1046/j.1365-313X.1999.00544.x 10476079

[B27] Gouvea C. F. Dornelas M. C. Rodriguez A. P. (2008). Floral development in the tribe cedreleae (meliaceae, sub-family swietenioideae): cedrela and toona. *Ann. Bot.* 101 39–48. 10.1093/aob/mcm279 17981877 PMC2701842

[B28] Greenberg J. T. (1997). Programmed cell death in plant-pathogen interactions. *Annu. Rev. Plant Physiol. Plant Mol. Bio.* 48 525–545. 10.1146/annurev.arplant.48.1.525 15012273

[B29] Guo J. Bai P. F. Yang Q. Liu F. R. Wang X. D. Huang L. L. (2013). Wheat zinc finger protein TaLSD1, a negative regulator of programmed cell death, is involved in wheat resistance against stripe rust fungus. *Plant Physiol. Biochem.* 71 164–172. 10.1016/j.plaphy.2013.07.009 23933226

[B30] Guo J. J. Liu C. Wang P. Cheng Q. Sun L. Yang W. C. (2018). The aborted microspores (AMS)-like gene is required for anther and microspore development in pepper (*Capsicum annuum* L.). *Int. J. Mol. Sci.* 19:1341. 10.3390/ijms19051341 29724052 PMC5983743

[B31] Guo W. J. Ho T. H. (2008). An abscisic acid-induced protein, HVA22, inhibits gibberellin-mediated programmed cell death in cereal aleurone cells. *Plant Physiol.* 147 1710–1722. 10.1104/pp.108.120238 18583533 PMC2492636

[B32] Han X. Lu M. Chen Y. Zhan Z. Cui Q. Wang Y. (2012). Selection of reliable reference genes for gene expression studies using real-time pcr in tung tree during seed development. *PLoS One* 7:e43084. 10.1371/journal.pone.0043084 22912794 PMC3422230

[B33] Hautegem T. V. Waters A. J. Goodrich J. Nowack M. K. (2015). Only in dying, life: programmed cell death during plant development. *Trends Plant Sci.* 20 102–113. 10.1016/j.tplants.2014.10.003 25457111

[B34] Huang H. Gao H. Liu B. Qi T. Tong J. Xiao L. (2017). *Arabidopsis* MYB24 regulates jasmonate-mediated stamen development. *Front. Plant Sci.* 8:1525. 10.3389/fpls.2017.01525 28928760 PMC5591944

[B35] Huang J. L. Gu M. Lai Z. B. Fan B. F. Shi K. Zhou Y. H. (2010). Functional analysis of the *Arabidopsis* PAL gene family in plant growth, development, and response to environmental stress. *Plant Physiol.* 153 1526–1538. 10.1104/pp.110.157370 20566705 PMC2923909

[B36] Kaltdorf M. Naseem M. (2013). How many salicylic acid receptors does a plant cell need? *Sci. Signal.* 6:jc3. 10.1126/scisignal.2003944 23757021

[B37] Kovacs J. Poor P. Szepesi A. Tari I. (2016). Salicylic acid induced cysteine protease activity during programmed cell death in tomato plants. *Acta Biol. Hung.* 67 148–158. 10.1556/018.67.2016.2.3 27165526

[B38] Langfelder P. Horvath S. (2008). WGCNA: an R package for weighted correlation network analysis. *BMC Bioinformatics* 9:559. 10.1186/1471-2105-9-559 19114008 PMC2631488

[B39] Li W. Liu M. Zhang L. Tan X. Zhang F. Wang Z. (2018). Study of major economic traits in 4 superior families of tung tree. *Non Wood For. Res.* 36 29–34. 10.14067/j.cnki.1003-8981.2018.02.005

[B40] Li Y. R. Williams B. Dickman M. (2017). *Arabidopsis* B-cell lymphoma2 (Bcl-2)-associated athanogene 7 (BAG7)-mediated heat tolerance requires translocation, sumoylation and binding to WRKY29. *New Phytol.* 214 695–705. 10.1111/nph.14388 28032645

[B41] Liao T. Yuan D. Y. Zou F. Gao C. Yang Y. Zhang L. (2014). Self-sterility in *Camellia oleifera* may be due to the prezygotic late-acting self-incompatibility. *PloS One* 9:e99639. 10.1371/journal.pone.0099639 24926879 PMC4057179

[B42] Livak K. J. Schmittgen T. D. (2001). Analysis of relative gene expression data using real-time quantitative PCR and the 2-ΔΔCT Method. *Methods* 25 402–408. 10.1006/meth.2001.1262 11846609

[B43] Lu Z. G. Xu J. Li W. X. Zhang L. Cui J. W. He Q. S. (2017). Transcriptomic analysis reveals mechanisms of sterile and fertile flower differentiation and development in *Viburnum macrocephalum* f. *keteleeri*. *Front. Plant Sci.* 8:261. 10.3389/fpls.2017.00261 28298915 PMC5331048

[B44] Mao Y. J. Liu W. B. Chen X. Xu Y. Lu W. L. Hou J. Y. (2017). Flower development and sex determination between male and female flowers in *Vernicia fordii*. *Front. Plant Sci.* 8:1291. 10.3389/fpls.2017.01291 28775735 PMC5517574

[B45] Mauchmani B. Slusarenko A. J. (1996). Production of salicylic acid precursors is a major function of phenylalanine ammonia-lyase in the resistance of *Arabidopsis* to *Peronospora parasitica*. *Plant Cell* 8 203–212. 10.1105/tpc.8.2.203 12239383 PMC161092

[B46] McCann L. P. (1942). Development of the pistillate flower and structure of the fruit of tung (*Aleurites fordii*). *J. Agr. Res.* 65:361.

[B47] Meininghaus R. Gunnarsen L. Knudsen H. N. (2000). Diffusion and sorption of volatile organic compounds in building materials-impact on indoor air quality. *Environ. Sci. Technol.* 34 3101–3108. 10.1021/es991291i

[B48] Millar A. A. Gubler F. (2005). The *Arabidopsis* GAMYB-like genes, MYB33 and MYB65, are microRNA-regulated genes that redundantly facilitate anther development. *Plant Cell* 17 705–721. 10.1105/tpc.104.027920 15722475 PMC1069693

[B49] Orosa B. He Q. Mesmar J. Gilroy E. M. Mclellan H. Yang C. (2017). BTB-BACK domain protein POB1 suppresses immune cell death by targeting ubiquitin E3 ligase PUB17 for degradation. *PLoS Genet.* 13:e1006540. 10.1371/journal.pgen.1006540 28056034 PMC5249250

[B50] Ostergaard L. Petersen M. Mattsson O. Mundy J. (2002). An *Arabidopsis* callose synthase. *Plant Mol. Biol.* 49 559–566. 10.1023/A:1015558231400 12081364

[B51] Pacini E. (1997). Tapetum character states analytical keys for tapetum types and activitie. *Can. J. Bot.* 75 1448–1459. 10.1139/b97-859

[B52] Pan Q. Han X. Bai Y. Yang J. (2002). Advances in physiology and ecology studies on stored non-structure carbohydrates in plants. *Chin. Bull. Bot.* 19 30–38.

[B53] Pearce S. Ferguson A. King J. Wilson Z. A. (2015). FlowerNet: a gene expression correlation network for anther and pollen development. *Plant Physiol.* 167 1717–1730. 10.1104/pp.114.253807 25667314 PMC4378160

[B54] Renner S. Feil J. P. (1993). Pollinators of tropical dioecious angiosperms. *Am. J. Bot.* 80 1100–1107. 10.2307/2445757

[B55] Rocheta M. Sobral R. Magalhaes J. Amorim M. I. Ribeiro T. Pinheiro M. (2014). Comparative transcriptomic analysis of male and female flowers of monoecious *Quercus suber*. *Front. Plant Sci.* 5:599. 10.3389/fpls.2014.00599 25414713 PMC4222140

[B56] Rogers H. J. (2006). Programmed cell death in floral organs: how and why do flowers die? *Ann. Bot.* 97 309–315. 10.1093/aob/mcj051 16394024 PMC2803638

[B57] Ruzin S. E. (2000). Plant microtechnique and microscopy. *New Phytol.* 148 57–58.

[B58] Sala K. Malarz K. Barlow P. W. Kurczynska E. U. (2017). Distribution of some pectic and arabinogalactan protein epitopes during *Solanum lycopersicum* (L.) adventitious root development. *BMC Plant Biol.* 17:25. 10.1186/s12870-016-0949-3 28122511 PMC5267361

[B59] Shannon P. Markiel A. Ozier O. Baliga N. S. Wang J. T. Ramage D. (2003). Cytoscape: a software environment for integrated models of biomolecular interaction networks. *Genome Res.* 13 2498–2504. 10.1101/gr.1239303 14597658 PMC403769

[B60] Shi Z. Maximova S. Liu Y. Verica J. Guiltinan M. J. (2013). The salicylic acid receptor npr3 is a negative regulator of the transcriptional defense response during early flower development in *Arabidopsis*. *Mol. Plant* 6 802–816. 10.1093/mp/sss091 22986789

[B61] Sobral R. Silva H. G. Morais-Cecilio L. Costa M. M. (2016). The quest for molecular regulation underlying unisexual flower development. *Front. Plant Sci.* 7:160. 10.3389/fpls.2016.00160 26925078 PMC4759290

[B62] Song Y. P. Ma K. F. Ci D. Chen Q. Q. Tian J. X. Zhang D. Q. (2013). Sexual dimorphic floral development in dioecious plants revealed by transcriptome, phytohormone, and DNA methylation analysis in *Populus tomentosa*. *Plant Mol. Biol.* 83 559–576. 10.1007/s11103-013-0108-2 23860796

[B63] Tao Q. Niu H. Wang Z. Zhang W. Wang H. Wang S. (2018). Ethylene responsive factor ERF110 mediates ethylene-regulated transcription of a sex determination-related orthologous gene in two *Cucumis* species. *J. Exp. Bot.* 69 2953–2965. 10.1093/jxb/ery128 29659946

[B64] Torres M. A. Dangl J. L. Jones J. D. (2002). *Arabidopsis* gp91phox homologues AtrbohD and AtrbohF are required for accumulation of reactive oxygen intermediates in the plant defense response. *Proc. Natl. Acad. Sci. U.S.A.* 99 517–522. 10.1073/pnas.012452499 11756663 PMC117592

[B65] Tsakas M. P. Siskos A. P. Siskos P. A. (2011). “Indoor Air Pollutants and the Impact on Human Health,” in *Chemistry, Emission Control, Radioactive Pollution and Indoor Air Quality* ed. Nicolas M. (London: IntechOpen) 447–484.

[B66] Ubeda-Tomas S. Bennett M. J. (2010). Plant development: size matters, and it’s all down to hormones. *Curr. Biol.* 20 R511–R513. 10.1016/j.cub.2010.05.013 20620902

[B67] Uppalapati S. R. Ishiga Y. Wangdi T. Kunkel B. N. Anand A. Mysore K. S. (2007). The phytotoxin coronatine contributes to pathogen fitness and is required for suppression of salicylic acid accumulation in tomato inoculated with *Pseudomonas* syringae pv. tomato DC3000. *MPMI* 20 955–965. 10.1094/MPMI-20-8-0955 17722699

[B68] Wan J. Patel A. Mathieu M. Kim S. Y. Xu D. Stacey G. (2008). A lectin receptor-like kinase is required for pollen development in *Arabidopsis*. *Plant Mol. Biol.* 67 469–482. 10.1007/s11103-008-9332-6 18392777

[B69] Wang D. Amornsiripanitch N. Dong X. (2006). A genomic approach to identify regulatory nodes in the transcriptional network of systemic acquired resistance in plants. *PLoS Pathog.* 2:e123. 10.1371/journal.ppat.0020123 17096590 PMC1635530

[B70] Wei W. J. Zhang Y. P. Xiong J. Y. Li M. (2012). A standard reference for chamber testing of material VOC emissions: design principle and performance. *Atmos. Environ.* 47 381–388. 10.1016/j.atmosenv.2011.10.051

[B71] Wildermuth M. C. Dewdney J. Wu G. Ausubel F. M. (2001). Isochorismate synthase is required to synthesize salicylic acid for plant defense. *Nature* 414 562–565. 10.1038/417571a 11734859

[B72] Wu H. M. Cheun A. Y. (2000). Programmed cell death in plant reproduction. *Plant Mol. Biol.* 44 267–281. 10.1023/A:1026536324081 11199388

[B73] Xiang X. Cao J. S. Ye W. Z. Cui H. M. Yu J. N. (2007). Molecular cloning and characterization of BcMYBogu, a novel member of the MYB family involved in OguCMS in *Brassica campestris* ssp. *chinensis*. *Hereditas* 5 621–628. 10.1360/yc-007-0621 17548334

[B74] Xu E. J. Brosche M. (2014). Salicylic acid signaling inhibits apoplastic reactive oxygen species signaling. *BMC Plant Biol.* 14:155. 10.1186/1471-2229-14-155 24898702 PMC4057906

[B75] Yamada M. Takeno K. (2014). Stress and salicylic acid induce the expression of PnFT2 in the regulation of the stress-induced flowering of *Pharbitis nil*. *J. Plant Physiol.* 171 205–212. 10.1016/j.jplph.2013.07.005 23973406

[B76] Yang T. Shad Ali G. Yang L. Du L. Reddy A. S. Poovaiah B. W. (2010). Calcium/calmodulin-regulated receptor-like kinase CRLK1 interacts with MEKK1 in plants. *Plant Signal Behav.* 5 991–994. 10.1074/jbc.M109.035659 20724845 PMC3115177

[B77] Yang Y. Liao Y. W. Tan X. F. (2018). Discussion on coordinated development of *Vernicia fordii* industry and future environment-friendly coatings industry in China. *Non Wood For. Res.* 36 188–192. 10.14067/j.cnki.1003-8981.2018.04.030

[B78] Zhao D. Z. Wang G. F. Speal B. Ma H. (2002). The excess microsporocytes1 gene encodes a putative leucine-rich repeat receptor protein kinase that controls somatic and reproductive cell fates in the *Arabidopsis* anther. *Genes Dev.* 16 2021–2031. 10.1101/gad.997902 12154130 PMC186413

[B79] Zhao Q. Zhou L. J. Liu J. C. Cao Z. Z. Du X. X. Huang F. D. (2018). Involvement of CAT in the detoxification of HT-induced ROS burst in rice anther and its relation to pollen fertility. *Plant Cell Rep.* 37 741–757. 10.1007/s00299-018-2264-y 29464319

[B80] Zhu J. Zhang G. Q. Chang Y. H. Li X. C. Yang J. Huang X. Y. (2010). AtMYB103 is a crucial regulator of several pathways affecting *Arabidopsis* anther development. *Sci. China Life Sci.* 53 1112–1122. 10.1007/s11427-010-4060-y 21104372

