# Supplementary figures and images for: New Insights of Salicylic Acid Into Stamen Abortion of Female Flowers in Tung Tree (Vernicia fordii)
Source: Front Genet. 2019 Apr 5;10:316. doi: 10.3389/fgene.2019.00316 (PMC6460477; doi:10.3389/fgene.2019.00316)

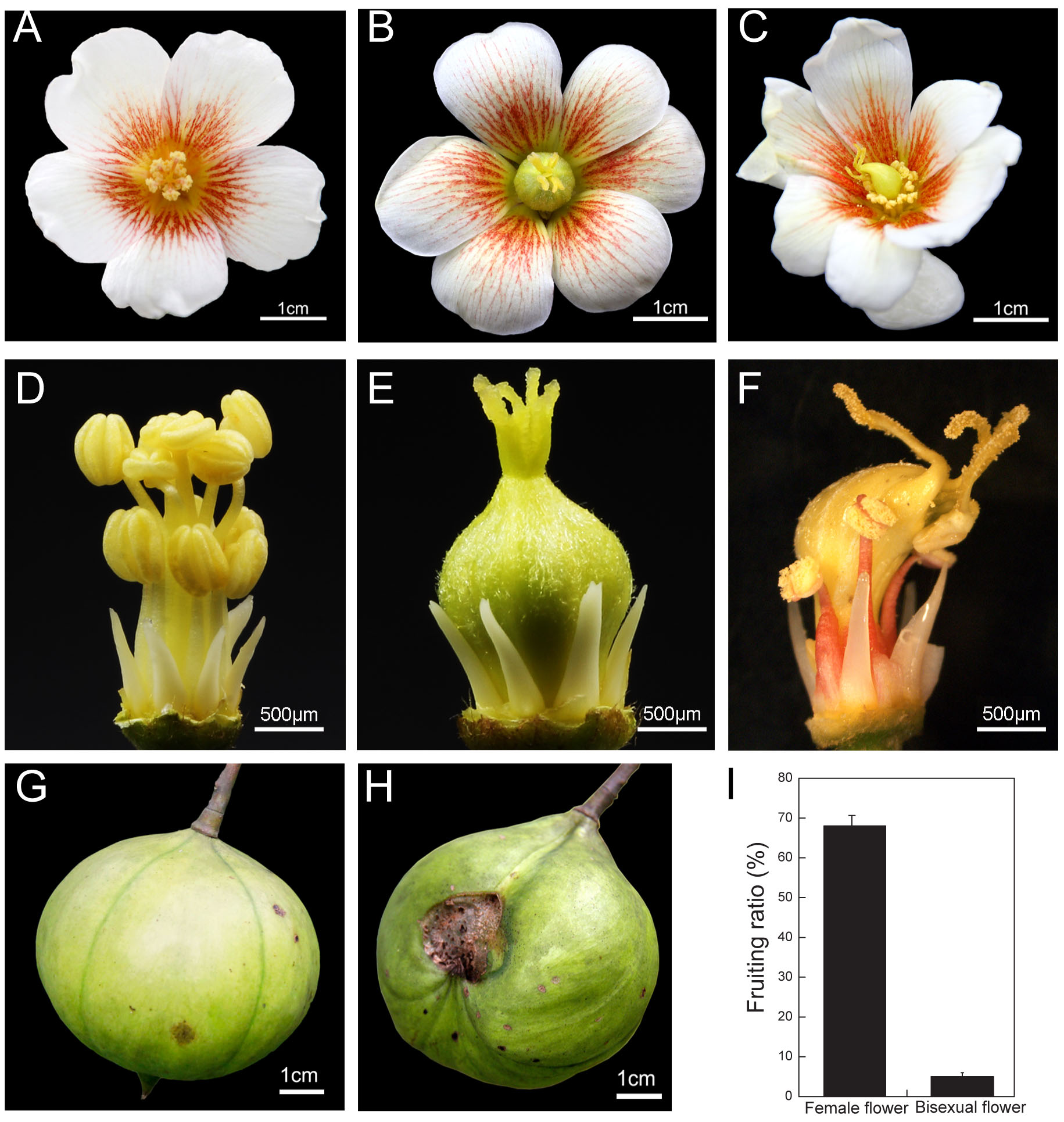

Supplement: FIGURE S1 — Tung tree flower and fruit. (A,D) Male flower. (B,E) Female flower. (C,F) Bisexual flower. (G,H) Tung fruit of female and bisexual flowers. (I) Fruiting ratio of female and bisexual flowers. [file Image_1.JPEG]
